# Barriers to GeneXpert utilization for tuberculosis detection at a regional referral hospital in Malawi: a qualitative study

**DOI:** 10.11604/pamj.2025.50.59.31398

**Published:** 2025-02-24

**Authors:** Jonathan Geophrey Majamanda, Mina Christine Hosseinipour, Maganizo Brave Chagomerana, Pascalia Munyewende, Ntombizodwa Ndlovu

**Affiliations:** 1 School of Public Health, Faculty of Health Sciences, University of the Witwatersrand, Johannesburg, South Africa,; 2 Malawi Adventist University, Malamulo College of Health Sciences, Thyolo, Malawi,; 3 University of North Carolina at Chapel Hill, Chapel Hill, North Carolina, USA,; 4 UNC Project-Malawi, Lilongwe, Malawi

**Keywords:** TB, Xpert-MTB/RIF, healthcare workers, diagnosis, suboptimal utilization

## Abstract

**Introduction:**

GeneXpert is the recommended diagnostic test for HIV-associated tuberculosis (TB). However, GeneXpert utilization is suboptimal in many countries. We explored the utilization of GeneXpert in an urban, regional referral hospital in northern Malawi using qualitative methods.

**Methods:**

in this cross-sectional qualitative study, a purposive sample of eight key informants from the TB clinic and laboratory was selected from outpatient and inpatient wards. An interview guide was used to conduct in-depth interviews to explore barriers to GeneXpert utilization. Interview data were analyzed using thematic analysis.

**Results:**

barriers to GeneXpert utilization appeared in three main themes: healthcare providers, institutions and operational-related factors. Healthcare provider factors included inadequate knowledge and training on differences in eligibility criteria for testing and GeneXpert algorithms by clinicians and laboratory technicians and poor interdepartmental communication. Institutional factors included staff shortages, heavy workloads and financial constraints. Operational factors included technical factors, e.g. power interruptions, GeneXpert module failures and poor sample quality and the restrictiveness of the algorithm.

**Conclusion:**

the study identified multiple factors that lead to GeneXpert underutilization. GeneXpert-specific training is required to address many of the provider-related barriers to utilization. This study highlighted the importance of assessing context-specific barriers to inform interventions to improve GeneXpert utilization.

## Introduction

Tuberculosis (TB) continues to be a leading cause of death due to a single infectious agent (second to COVID-19) despite substantial public health efforts to combat its spread [1]. Central in the fight against TB is improved case detection [2].

With improved testing methods and cost-effective and accurate case detection, there can be timely treatment initiation and hence higher recovery rates of patients with TB. Deficiencies in TB diagnosis contribute to the ongoing TB burden in lower and middle-income countries (LMICs). For instance, smear microscopy detects only 36-43% of TB cases and chest radiographs are costly and require expertise to read and interpret the findings [3,4]. The TB culture test has a long turnaround time of two to six weeks and requires significant laboratory infrastructure, which is often not available in rural settings in LMICs [3,5].

In 2010, the World Health Organization (WHO) endorsed the GeneXpert, also known as Xpert-MTB/RIF, assay as the initial diagnostic test for people presumed to have multi-drug resistant (MDR) or HIV-associated TB [6]. Its use has resulted in significant improvements in case detection, notification and patient waiting times [7,8]. GeneXpert is a molecular nucleic acid amplification test that recognizes a specific gene in TB bacteria and amplifies it to detectable levels [2]. The test produces results in two hours, thus ensuring timely treatment by enabling patients to have same-day disease detection and treatment initiation [9,10]. GeneXpert is simple to run and is particularly useful in high HIV-TB burden countries since it can be used at near point-of-care and to detect drug-resistant strains of mycobacteria [11,12].

In 2015, the WHO reported that 41% of new TB cases were not detected despite the availability of GeneXpert at the testing sites [13]. A systematic review covering 17 countries (Ethiopia, Nigeria, South Africa, Zambia, Zimbabwe, Bangladesh, Cambodia, India, Nepal, Pakistan, Brazil, Colombia, Democratic Republic of Congo, Kenya, Moldova, Mozambique and Malawi) reported average turnaround times ranging from one to seven days. It has also been shown that although most health facilities had laboratories for GeneXpert testing, only 4% of HIV-TB co-infected patients were tested using GeneXpert due to cartridge stockouts and electricity interruptions [14]. Suboptimal GeneXpert utilization rates of between 8% and 63.5% were reported in Nigeria [15], Uganda [16] and Zimbabwe [17].

Malawi has a dual burden of HIV and TB. In Malawi, the estimated TB incidence has declined from 338 per 100,000 in 2010 to 132 per 100,000 in 2021 [18]. The decline may be partially explained by disruptions caused by the COVID-19 pandemic [19].

Early detection and treatment of TB remain important factors for TB control. Traditional methods of TB detection have been smear microscopy, chest radiography and clinical judgment [20]. The GeneXpert test, a real-time PCR-based rapid molecular assay for rapid, early and improved detection of TB, was rolled out to central regional referral and district hospitals in Malawi in 2011 with coverage limited to selected district, community and private hospitals [21]. Challenges associated with its use include poor turnaround times and suboptimal utilization [21]. Studies conducted in Malawi on healthcare workers involved in the TB programme [22] and patients [23] identified institutional, operational, client, and healthcare provider factors that caused the underutilization of GeneXpert. This study focused on clinicians and laboratory staff to identify barriers to GeneXpert utilization in a hospital in Malawi. The findings will inform interventions to improve GeneXpert uptake in healthcare facilities and contribute to more accurate and early TB diagnosis, resulting in timely initiation of anti-TB treatment towards achievement of the Stop TB strategy [24].

## Methods

### Study design and setting

In this cross-sectional study, we used the grounded theory qualitative research design to provide in-depth understanding of GeneXpert underutilization and to identify associated barriers. The study was conducted at one of the four regional referral hospitals in Malawi. The hospital was chosen because it had a low GeneXpert utilization rate despite the high TB prevalence in the population that it serves. The hospital laboratory is the only urban referral laboratory in the Northern Region of Malawi and serves approximately 221,000 people in one of the country´s largest cities by population, and 2.3 million people in the region [25]. The setup and functioning of the laboratory were similar to those of other central regional referral hospital laboratories in Malawi. The TB laboratory is nested within the hospital´s general laboratory. All laboratory technicians are oriented in TB diagnostic methods and are rotated through the laboratory, including the TB section, to ensure that they can work in any section.

### Participant recruitment

Purposive sampling was used to identify eligible participants. Clinicians and laboratory technicians who had undergone GeneXpert training, with at least five years of work experience and who were working in the TB clinic or laboratory at the time of the study, were invited telephonically to participate in the study. All invited participants agreed to participate.

### Data collection

An interview guide with eight open-ended questions was developed and piloted before the study to assess clarity of questions and length of interviews. Leading questions and probes identified in the pilot were removed and some questions were rephrased to improve clarity. The interview guide had eight open-ended questions that assessed knowledge about GeneXpert, experiences in TB patient and sample management for GeneXpert testing, procedures followed to ascertain patient eligibility for GeneXpert testing, challenges experienced while using GeneXpert, attendance of comprehensive GeneXpert training and factors associated with utilization.

In-depth interviews (IDIs) were conducted in February 2018. All interviews were conducted in English and were audio recorded. The interviews were conducted at the hospital in comfortable, secluded spaces chosen by the participating healthcare workers using the interview guide. The IDIs lasted between 30 and 45 minutes per individual. While in the field, the interviewer recorded keynotes for reference. No repeat interviews were conducted.

### Data analysis

We used Braun and Clarke's (2006) contemporary approach to reflexive thematic analysis [26]. The recordings were transcribed and checked by the researcher. A codebook was developed as themes appeared during thematic analysis in Nvivo11 QSR International. Two transcripts were coded independently by two individuals (JGM and PM). The codes were compared and discussed. Agreed codes were used to code the remainder of the transcripts. Only themes that were relevant to the research question were considered for analysis.

### Researcher characteristics

At the time of the study, the principal investigator, JGM, was a full-time MSc Epidemiology (Implementation Science) student and conducted this study in partial fulfillment of his degree. He has a medical laboratory science background and had worked in the hospital where this study took place as a laboratory technologist. He had worked in various laboratory departments, including TB, chemistry and haematology. He introduced himself to participants as a former worker at this hospital and conducted the interviews in any of the three local languages: Tumbuka, Chewa and English. This made participants comfortable to answer questions and to express themselves in the language of their choice.

### Ethics consideration

Ethics clearance was obtained from the University of Witwatersrand Human Research Ethics Committee (Medical) (Number: M171193) and the Malawi National Health Sciences Research Committee (Number: 17/11/1920). Permission to conduct the study was obtained from hospital management. Written informed consent to participate in the study and for audio recording of the interviews was obtained from all participants before the interviews. No personal identifiers were recorded, and each participant was given a pseudonym.

## Results

Eight staff members met the eligibility criteria and consented to participate in the study. Three were laboratory technicians and five were clinicians. All participants were male. The ages of the participants ranged from 21 to 42 years. Three clinicians were 21-30 years old and two were in the 31-40-year age group. There was one laboratory technician in each of the three age groups (21-30 years, 31-40 years and >40 years).

One laboratory technician represented the University Research Company (URC), an organization that offered technical support to medical laboratories. Three of the clinicians worked as clinical associates and two were medical doctors. In terms of education, one laboratory technician had a diploma, one a degree and one a postgraduate degree. Three clinicians held diplomas and two had degrees. Two laboratory technicians and two clinicians had 5-10 years of experience working in TB laboratories or clinics, and one laboratory technician and three clinicians had over 10 years of experience.

In the IDIs, participants were thoughtful and keen to share their ideas. The three themes that emerged as barriers to GeneXpert utilization were grouped under the themes: health provider, institutional and operational factors. The themes are summarized in Table 1.

**Table 1:** summary of barriers to GeneXpert utilization

Theme	Description
Healthcare provider factors	
Knowledge	- Inadequate knowledge of GeneXpert- Poor knowledge of discrepancies between clinic and laboratory GeneXpert algorithms and eligibility criteria
Communication	-Poor collaboration and communication between clinical and laboratory departments-Poor instruction of patients on sputum specimen production-Unpleasantness of working with sputum specimens
Training	- TB training with inadequate GeneXpert training- Same staff attend training
Institutional factors	
Staffing	-Staff shortages and heavy workloads
Financial resources	- Insufficient financial resources to support GeneXpert testing resulting in use of microscopy- Restrictive algorithm related to limited resources
Operation factors	
Technical	- Reagent stockouts- GeneXpert module failures- Poor quality sputum specimens- Power failures
Algorithm	-Restrictive TB algorithm

### Healthcare provider factors

#### Knowledge

Several knowledge and information gaps for clinicians emerged. These included poor awareness and knowledge of the availability of GeneXpert and other TB diagnostic tests at the hospital. There was also poor knowledge of the GeneXpert test guidelines and discrepancies in the for clinicians and laboratory guidelines describing patient eligibility for testing. One clinician noted that the test selection algorithms for the clinical and laboratory departments were different. Hence, sometimes when clinicians ordered GeneXpert tests for specimens, the laboratory staff rejected the requests or diverted the specimens to microscopy.

*“…most of our samples that we send to the lab for GeneXpert are just done microscopy. We use different eligibility steps with them.” (Clinician-1)*.

#### Collaboration

Poor communication between the clinicians working in the hospital and laboratory staff led to information asymmetry as “*most clinicians don´t know much about the GeneXpert*” (Lab-technician-3). There was poor information sharing between the clinical and laboratory departments. This challenge could be avoided through improved interdepartmental communication.

*“There is lack of communication and interaction. If the laboratory guys can link with us on GeneXpert, we can have the information they know and the information the clinicians know and there cannot be these GeneXpert problems.” (Clinician-2)*

Most test request forms submitted to the laboratory by clinicians were incomplete or completed incorrectly. At times, test request forms were sent to the laboratory with missing information, such as patient name, age, inpatient or outpatient department (OPD), and HIV status. Laboratory technicians used this information to decide on the suitability of the specimen for GeneXpert testing using the laboratory test algorithm. Without complete information, the laboratory technicians could not conduct the requested tests, thus causing delays, increasing turnaround times and sample rejections.

*“Sometimes we are all stranded. We don´t know what to do. What test would we carry out on this particular sample because the form isn´t properly filled? It´s incomplete.” (Lab-technician-2)*

Failure by clinicians to provide patients with adequate sputum production instructions resulted in many patients producing insufficient sputum, resulting in specimen rejection by the laboratory. Some sputum specimens contained food particles, which could potentially block the GeneXpert machine, causing malfunctions and delays. The inability to instruct patients was attributed to inadequate GeneXpert and TB training.

*“The first challenge is poor information as the clinicians are not given full information on how the test works and how best they can do the orders…. because there is inadequate training on GeneXpert or TB.” As a result, “most clinicians are not very conversant with what to tell patients to produce quality sample.” (Clinician-5)*.

A laboratory technician raised a concern about working with sputa, which he found to be unpleasant resulting in delays in the processing of specimens for GeneXpert tests.

*“A lot of people don´t enjoy working with sputum. They may enjoy workshops but [laughs] on the ground they don´t enjoy working with sputum. So, we have a lot of people who have been trained, but very few who are interested. So, you find the few who are interested - the moment they are away - the samples cannot be handled for GeneXpert. So that´s the other key element which is lowering utilization and prolonging turnaround time.... I remember another time I was on holiday. I found samples - two weeks they are just there. So, you see how it feels.” (Lab technician-1)*

#### Training

The lack of focus on GeneXpert in TB workshops offered at the hospital was perceived to reduce its importance in testing. This led to the accumulation of TB specimens in the laboratory and increased turnaround times.

*“Most of this training is just TB training. There is no specific training for GeneXpert. So, this makes it appear as a less important test method.” (Clinician-2)*.

### Institutional factors

#### Staffing

Some participants observed that the shortage of doctors resulted in shortened doctor-consultation times. This provided insufficient time for comprehensive history-taking and led to poor diagnoses because clinicians missed some signs of TB. In addition, being in a regional referral hospital, the laboratory received many TB and non-TB referral samples, which created heavy workloads and increased turnaround times.

*“Sometimes the results take more than a week because we are a referral laboratory here in the Northern region. So, most of the sputum samples come here. So, it´s like big work for us” (Lab-technician-2)*.

#### Financial resources

The high cost of the GeneXpert test was considered a barrier to utilization. Although participants agreed that GeneXpert is superior to microscopy due to minimal sample manipulation, reduced time to obtain results and provision of detailed result profiles, microscopy is often ordered to reduce costs.

*“We order microscopy instead of GeneXpert although we know it´s not the best practice because microscopy has very low sensitivity when compared to GeneXpert. But what can we do?” (Clinician-4)*

Participants felt that limited resources were responsible for the government´s decision not to test all TB presumptive patients using GeneXpert. There was a perception that the eligibility criteria were designed to reduce the number of patients eligible for GeneXpert testing and therefore reduce costs.

*“I feel just because of the limited resources… that´s why the Malawi government came with the at-risk group to be tested on GeneXpert”. (Lab-technician-3)*

### Operational factors

#### Technical

All participants highlighted cartridge and reagent stockouts, GeneXpert module failures, poor quality sputum specimens, and power supply interruptions as causes for poor GeneXpert utilization and significant delays in specimen testing. At the time of the study, there had been fewer reagent stockouts.

*“Previously we could go for weeks without reagents. Now we are not having as many stockout challenges. I think we are much better…” (Lab-technician-3)*

Some laboratory technicians stated that electricity interruptions caused delayed GeneXpert testing. This was the case, especially when power interruptions lasted longer than the time that the uninterruptible power supply (UPS) batteries could provide power to run GeneXpert tests.

#### Algorithm

Participants also reported that the national TB algorithm was restrictive as it excluded some patients from GeneXpert testing. Only HIV-positive, MDR-TB presumptive cases, mine workers and inpatients were eligible for GeneXpert testing, thus excluding many TB presumptive cases who could have benefited from the diagnosis.

*“Since we adhere to just what has been stipulated there…you can have, per day 10 TB samples all requiring GeneXpert. But if these include eight HIV-negative patients and they are OPDs, only two will qualify for the GeneXpert. So that´s one of the key factors for underutilization.” (Lab-technician-1)*

## Discussion

In this study, we sought to identify factors that contributed to GeneXpert underutilization at a regional referral hospital in northern Malawi. Our study focused specifically on documenting the experiences of clinicians and laboratory staff who worked in the TB clinic or laboratory. Our findings are similar to those from Malawi and other LMICs that have identified several healthcare provider, institutional and operational factors that contribute to the underutilization of GeneXpert testing [22,23,27,28]. While the findings are not generalizable, they are relevant to other similar healthcare settings in Malawi and other countries that follow similar procedures for obtaining and testing TB specimens.

Poor knowledge and use of the clinical and laboratory TB testing algorithms and eligibility criteria contributed to underutilization of GeneXpert testing. An earlier study in Malawi [29] and another in Ethiopia [30] showed that over half of the healthcare workers studied (64% and 54.6%, respectively) studied had low knowledge about GeneXpert and eligibility criteria resulting in reduced utilization of the test. Agonifar *et al*. (2018) found that healthcare workers who had read the GeneXpert guidelines were more likely to use the test effectively, suggesting that higher knowledge leads to increased utilization [30]. The participants also noted that the algorithms themselves were restrictive, resulting in low numbers of patients eligible for testing. There is a need to streamline the GeneXpert guidelines so that eligibility criteria for testing used by clinicians and laboratory technicians are consistent.

Poor-quality sputum samples were also identified as a barrier. Similar to our findings, a study in Uganda found that patients were rarely informed about how to produce sputum of good quality [31]. The study highlighted the need to prioritize the training of healthcare workers on skills to communicate instructions effectively to reduce specimen rejection [31]. The laboratory also reported that they often received forms with missing or incorrect information from the TB clinic.

Laboratory staff must inform clinicians if specimens are ineligible for testing based on the laboratory algorithm, if sputum specimens are of poor quality, or if forms have not been completed satisfactorily. Good interdepartmental communication between clinicians and laboratory staff is needed to improve GeneXpert testing uptake and turnaround times.

Although the healthcare workers in our study had received TB training, it did not include comprehensive GeneXpert training. A similar observation was made by Agonafir *et al*. (2018), who found that only 46% of TB clinic staff had been trained on GeneXpert during TB training and that there were knowledge gaps that affected utilization of the test [30]. These authors suggested distribution of the national GeneXpert guideline to TB clinic workers and training to ensure understanding of the contents [30]. In our setting, tuberculosis training for healthcare workers should incorporate training on the content of GeneXpert algorithms, eligibility criteria, sputum collection and methods to improve communication and collaboration between departments in the hospital and between clinicians and patients. Training should also address the laboratory staff's concerns regarding the unpleasantness of working with sputa.

Clinicians noted that staff shortages and heavy workloads resulted in missed diagnoses of TB. Laboratory staff are overburdened by the large numbers of specimens submitted to the referral hospital for processing. These issues contribute to the underutilization of GeneXpert and are not unique to this hospital. It has been shown that the addition of human resources led to improved utilization in Mongolia and Pakistan [27]. In the fight against TB, healthcare worker shortages are a reality that needs to be tackled at government level not only in Malawi but in other African countries as well [32,33].

The high cost of running GeneXpert has also contributed to low GeneXpert utilization. While GeneXpert is a more efficient and sensitive test compared to microscopy, costs for running and maintenance, reagents and consumables, are high [34]. High costs in the face of declining donor support have made some hospitals in LMIC settings opt for microscopy and thereby hampered the scale-up of GeneXpert [34,35]. This preference has major implications for TB detection and there have been calls to reduce the cost of GeneXpert to enable its wider usage [34].

Reagent and cartridge stockouts, blackouts and module failures were recurring technical factors that reduce GeneXpert utilization and have also been reported in Uganda [28]. Improved GeneXpert functionality through timely maintenance and service, useful cartridge supply systems, and laboratory support systems are needed. In countries like Malawi where regular power outages occur, alternative sources of power should be explored. A systematic review to assess barriers and facilitators of implementation of GeneXpert for TB testing in LMICs identified power interruptions as one of several barriers to GeneXpert utilization in several countries (Mozambique, Nepal, Nigeria, Pakistan, Swaziland and Uganda) [27]. In Nigeria, batteries and inverters were utilized for backup power storage, and due to prolonged power outages that exceeded the capacity of the batteries, some laboratories installed solar panels [36].

A limitation associated with the cross-sectional study design used in this study is that the findings may not be generalizable. Although restricted to a single site, the study underscores the importance of understanding the local context to address challenges related to the implementation of GeneXpert and enable the development of context-specific interventions. Therefore, the approach used and insights might be useful in similar settings. A recent systematic review of GeneXpert utilization studies identified barriers similar to those identified in our study, viz. poor communication, inadequate training of staff, restricted testing algorithms for identification of eligible patients and stockouts [27]. However, in our study, we elaborated on context-specific bottlenecks within these broad categories.

Strengths of the study include that the participants were long-serving practitioners who had insight and experience in working in the TB clinic and laboratory. The use of in-depth interviews allowed participants to share information without being limited by the scope of the questions. As suggested by Brown *et al*. [27], the use of a theoretical framework to guide the development of interview guides and analysis, may have ensured that factors, both barriers and enablers, were explored more comprehensively. An example of a suitable framework that can be used in such studies is the Consolidated Framework for Implementation Research (CFIR), which has five domains that explore characteristics of the innovation, inner setting, outer setting, individuals involved, and implementation process [37].

## Conclusion

This study highlights several barriers to GeneXpert utilization. Our findings can be used to inform the development of interventions to improve knowledge and motivate health workers to use GeneXpert and to address some of the healthcare providers, institutional and operational barriers that reduce GeneXpert utilization in hospitals in Malawi and other similar settings.

### What is known about this topic

- The GeneXpert diagnostic tool was introduced to improve tuberculosis case detection, notification and testing turnaround times and contribute to the fight to combat the disease;
- In many settings, including Malawi, GeneXpert is underutilized, leading to suboptimal TB case detection.

### What this study adds

- Clinicians and laboratory staff identified several barriers to GeneXpert utilization within the main themes of institutional, operational and healthcare provider-related factors in an urban regional referral hospital in Malawi;
- These findings can be used to inform interventions to improve GeneXpert utilization in similar settings.
